# Supplementary figures and images for: 3D reconstruction enables high-throughput phenotyping and quantitative genetic analysis of phyllotaxy
Source: Plant Phenomics. 2025 Mar 8;7(1):100023. doi: 10.1016/j.plaphe.2025.100023 (PMC12710043; doi:10.1016/j.plaphe.2025.100023)

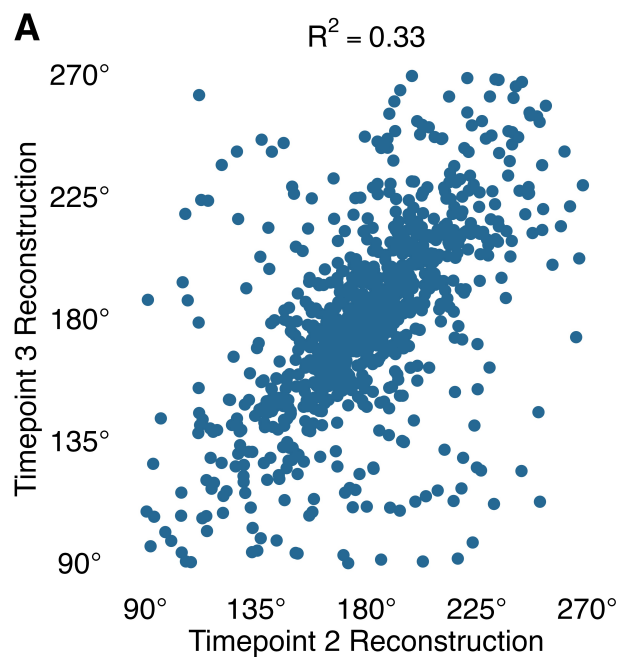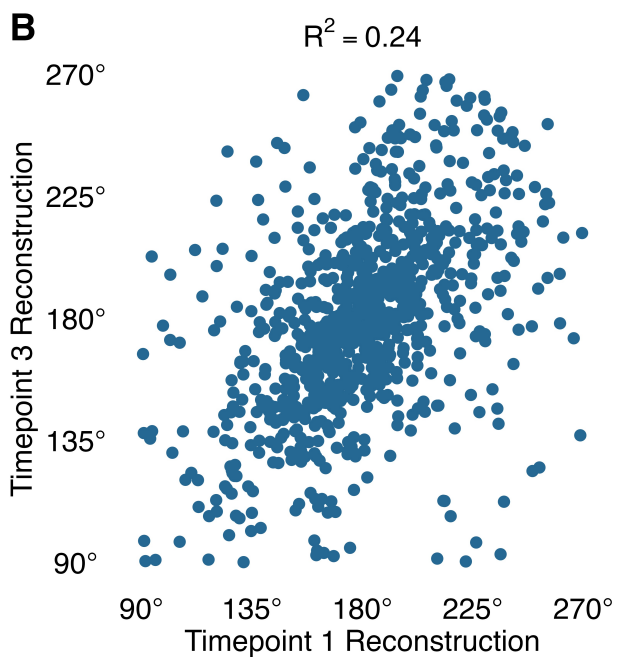

Supplement: Multimedia component 1 [file mmc1.pdf]

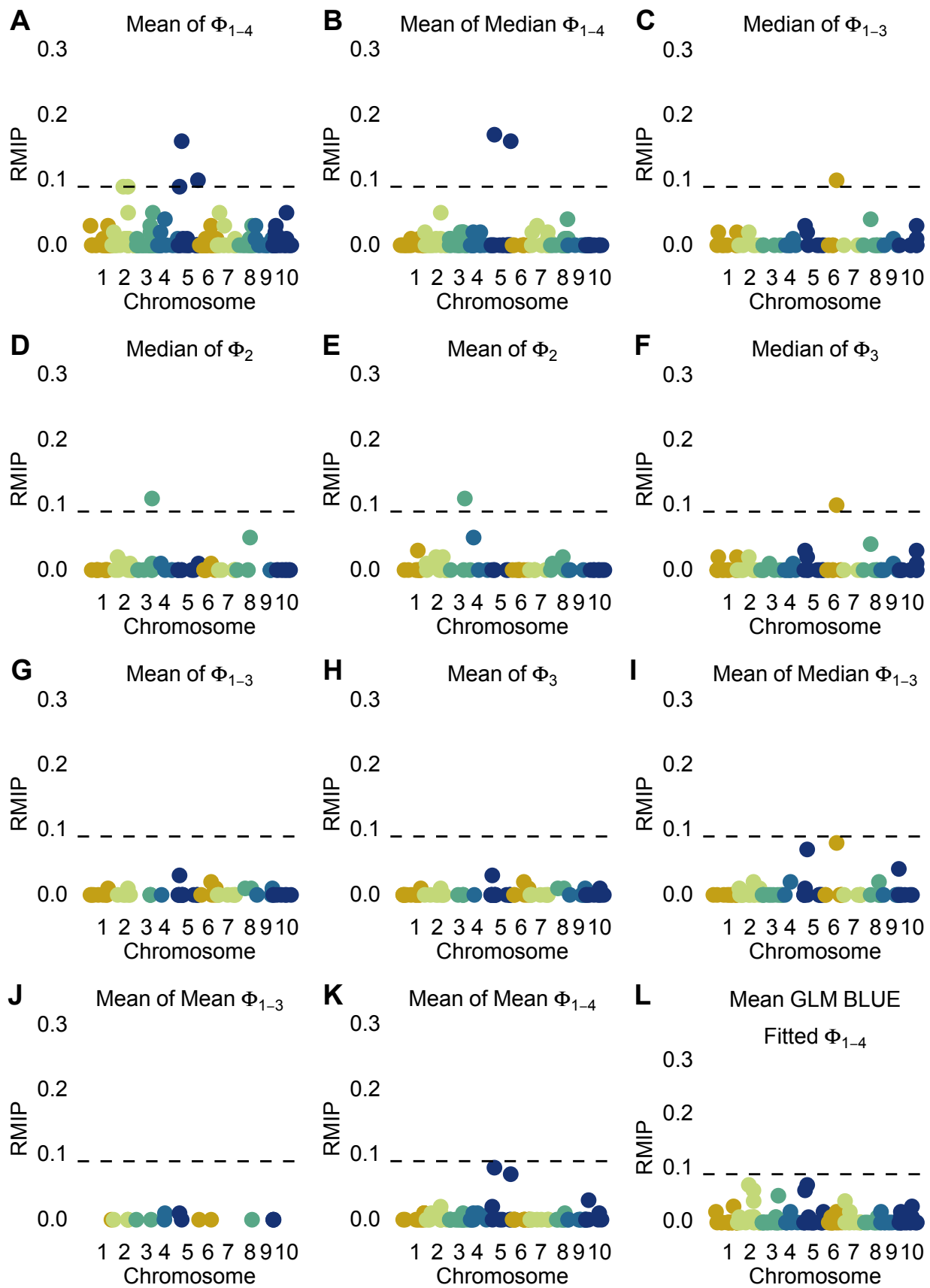

Supplement: Multimedia component 2 [file mmc2.pdf]

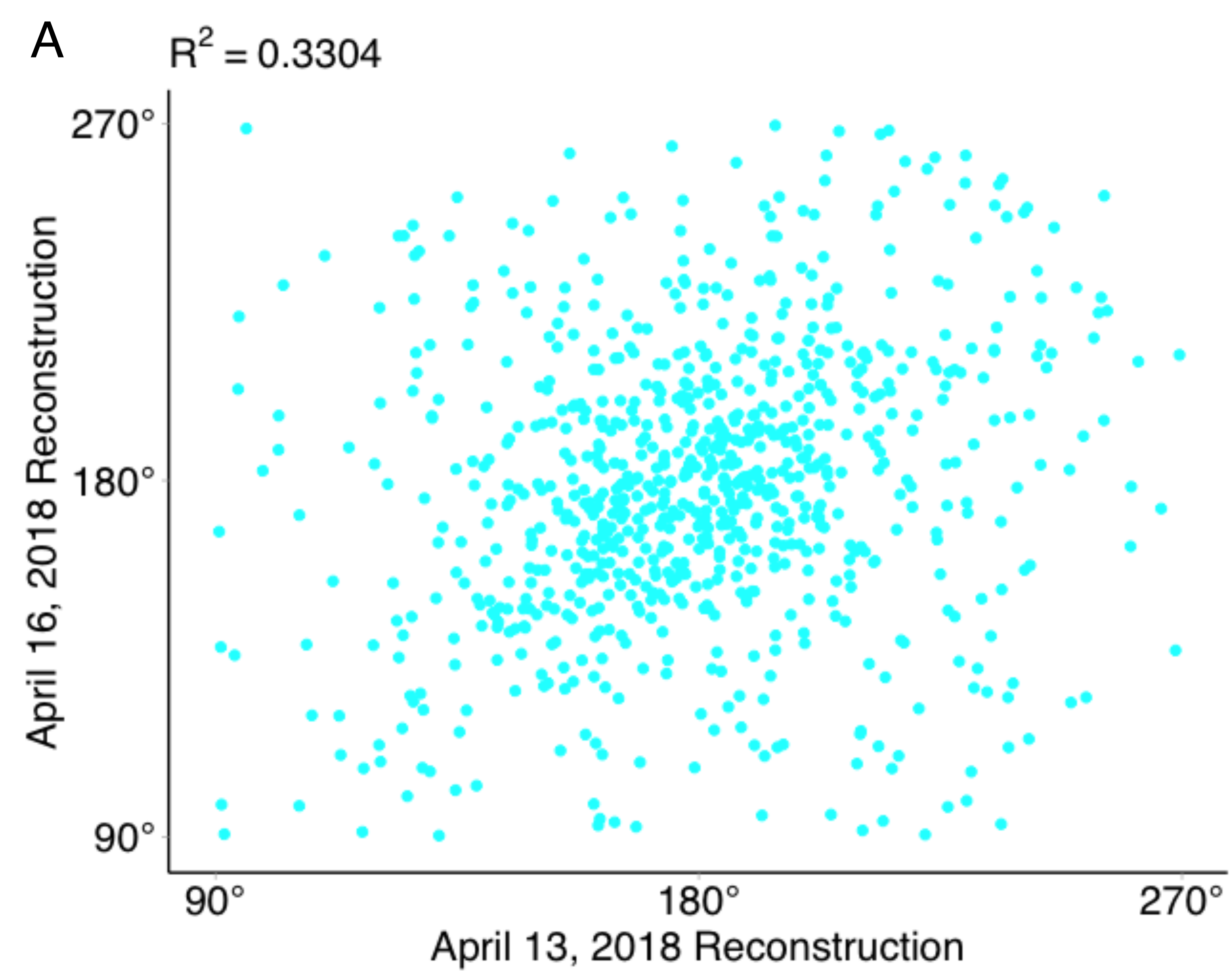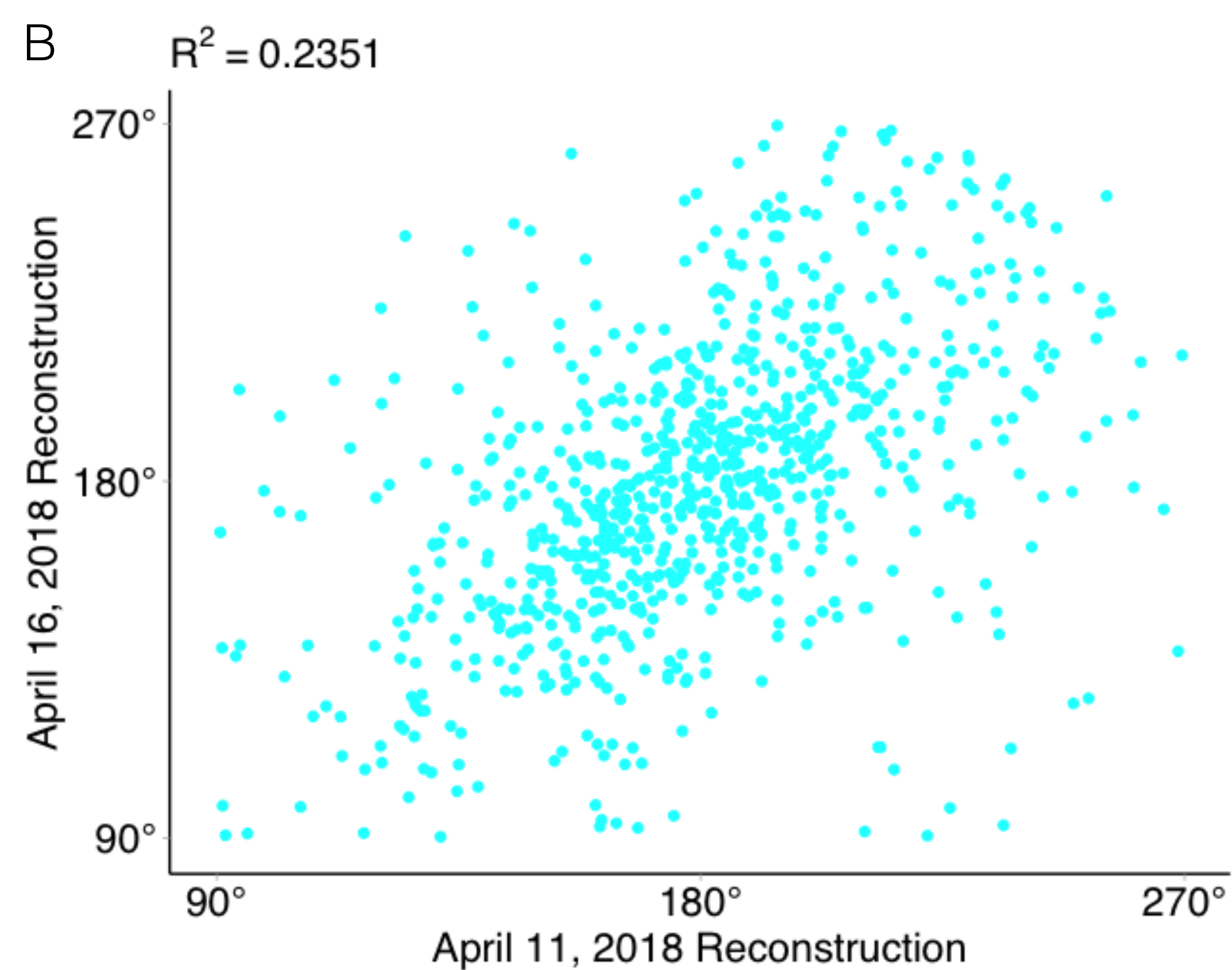

Supplement: Multimedia component 3 [file mmc3.zip › figs/Supplementary_reconstructionReliability.pdf]

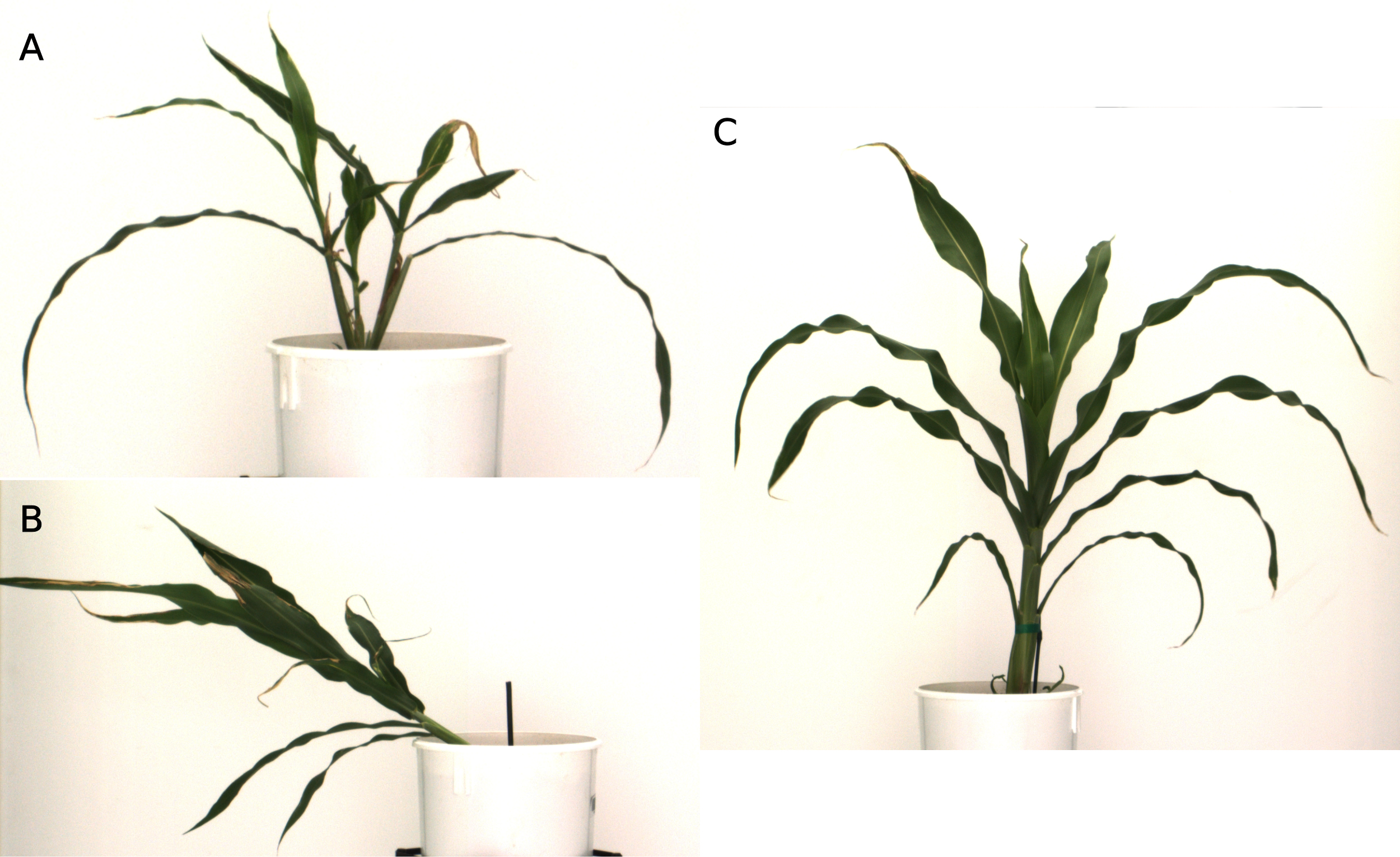

Supplement: Multimedia component 3 [file mmc3.zip › figs/tillers.jpg]

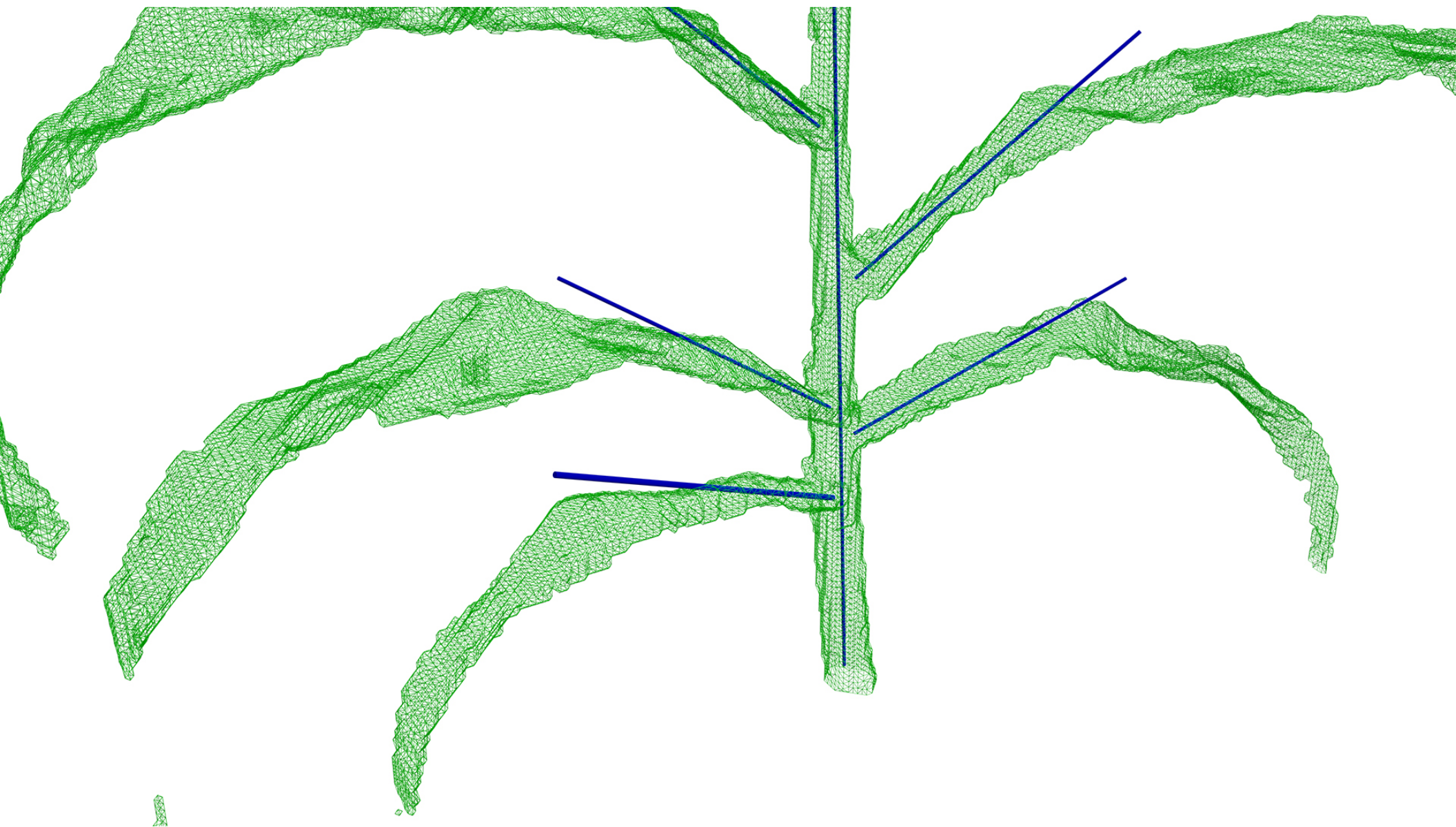

Supplement: Multimedia component 3 [file mmc3.zip › figs/fig_angle_rendering_fullhd.pdf]

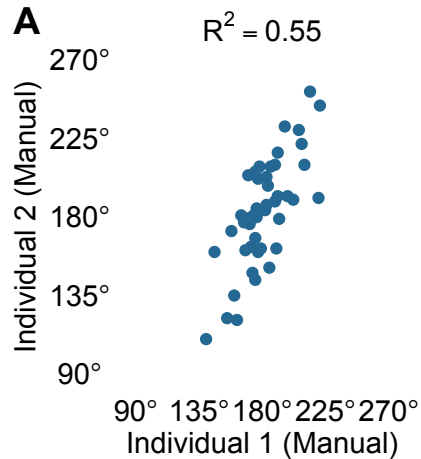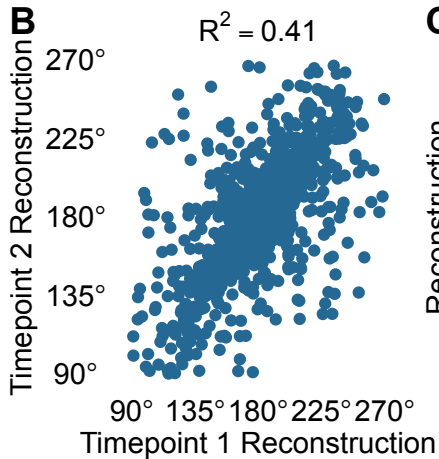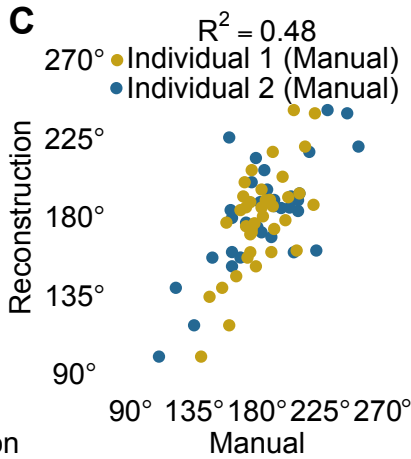

Supplement: Multimedia component 3 [file mmc3.zip › figs/reliabilityPlots.pdf]

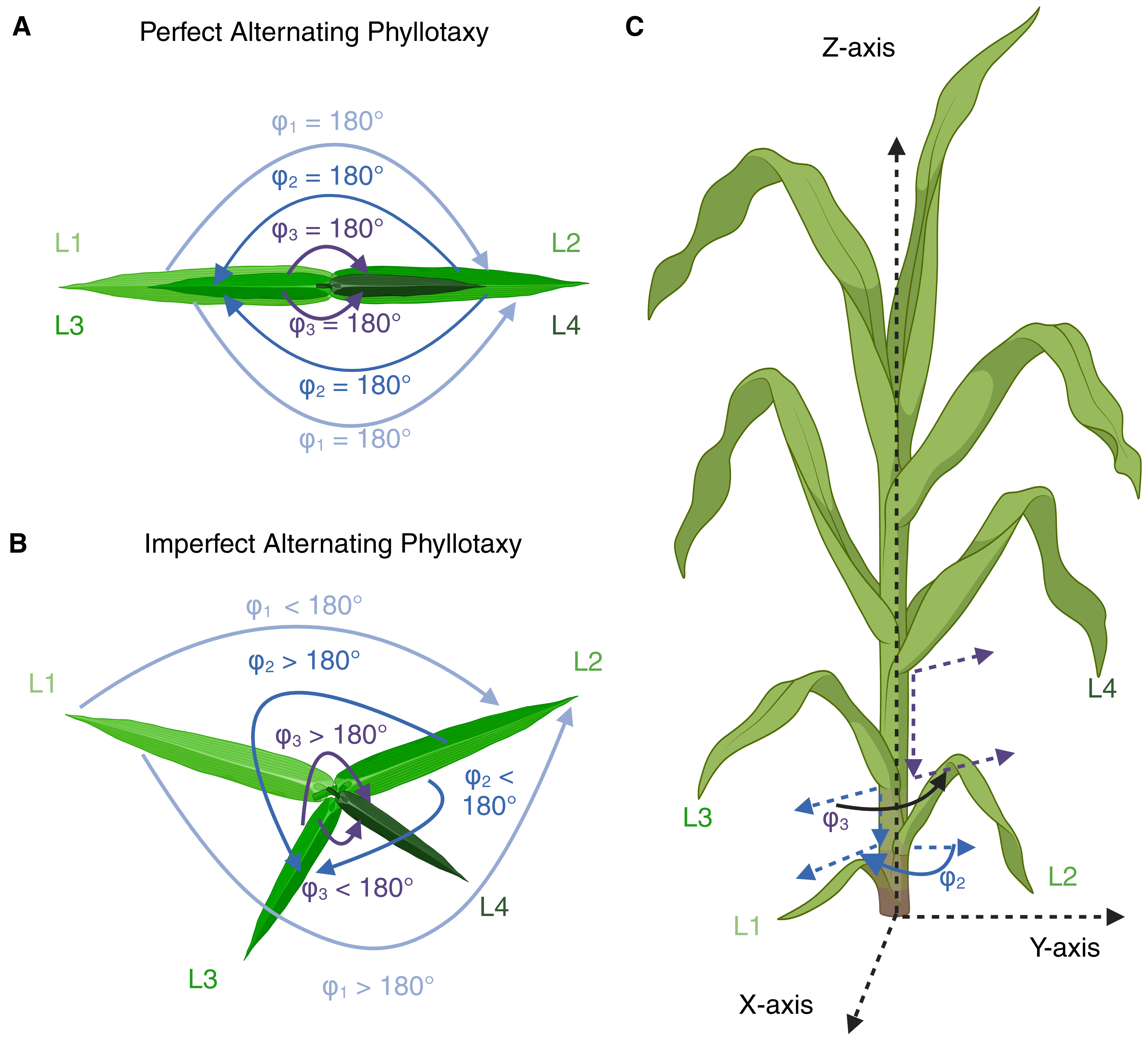

Supplement: Multimedia component 3 [file mmc3.zip › figs/Phyllotaxy_Conceptual_JDavis.png]

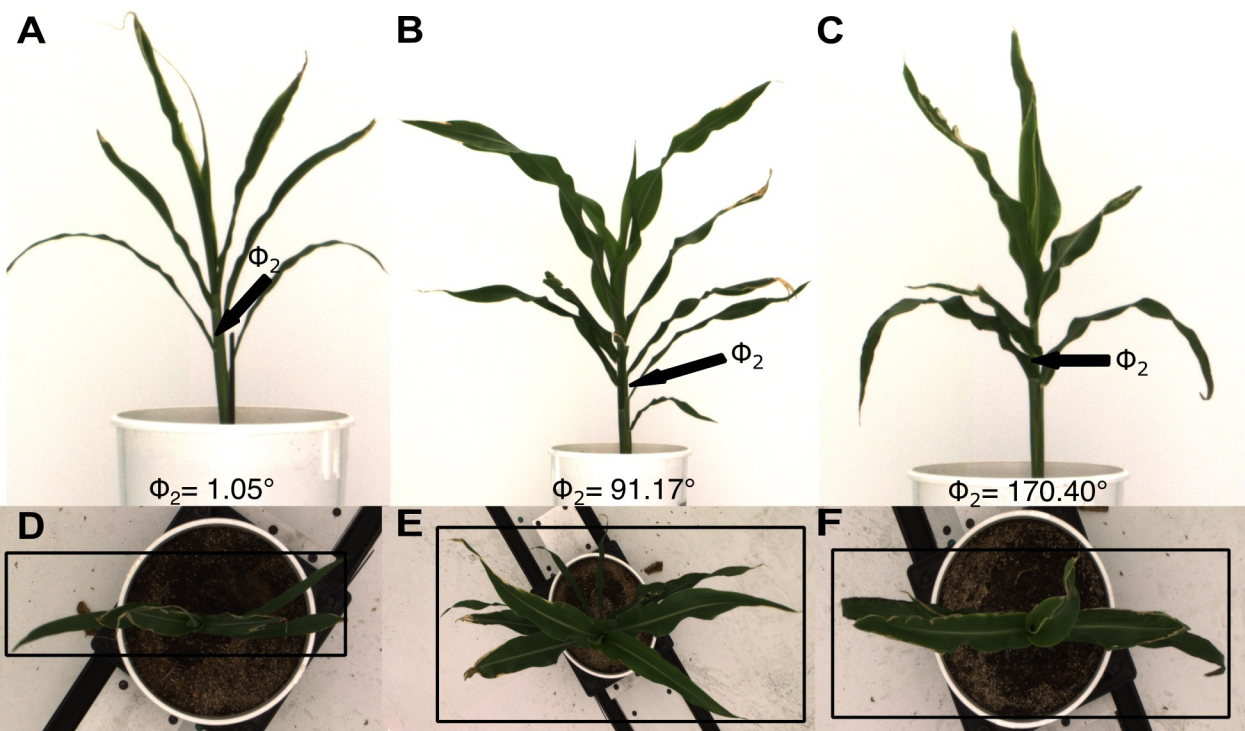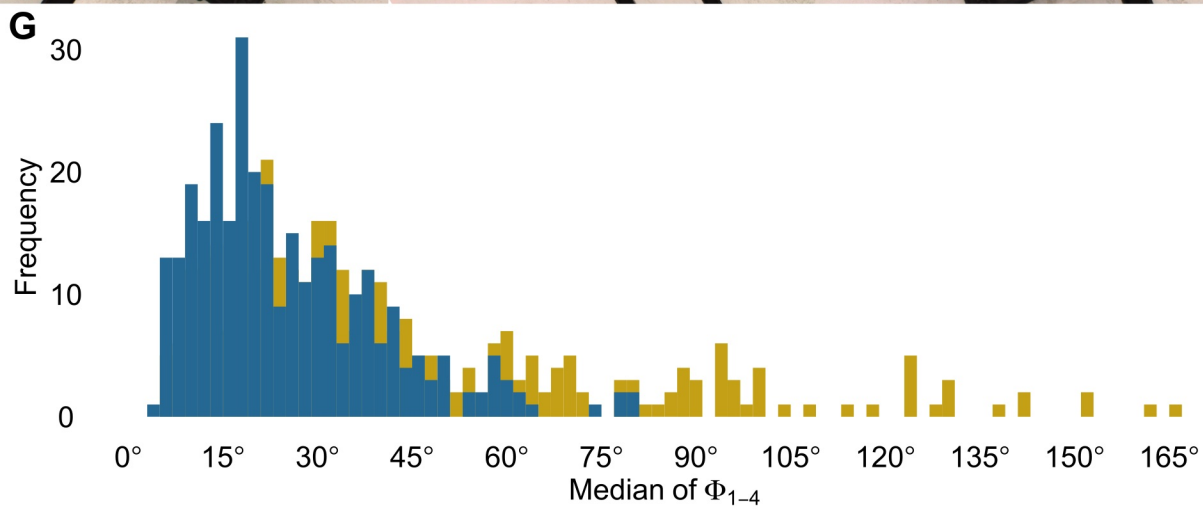

Supplement: Multimedia component 3 [file mmc3.zip › figs/phylloVariation.pdf]

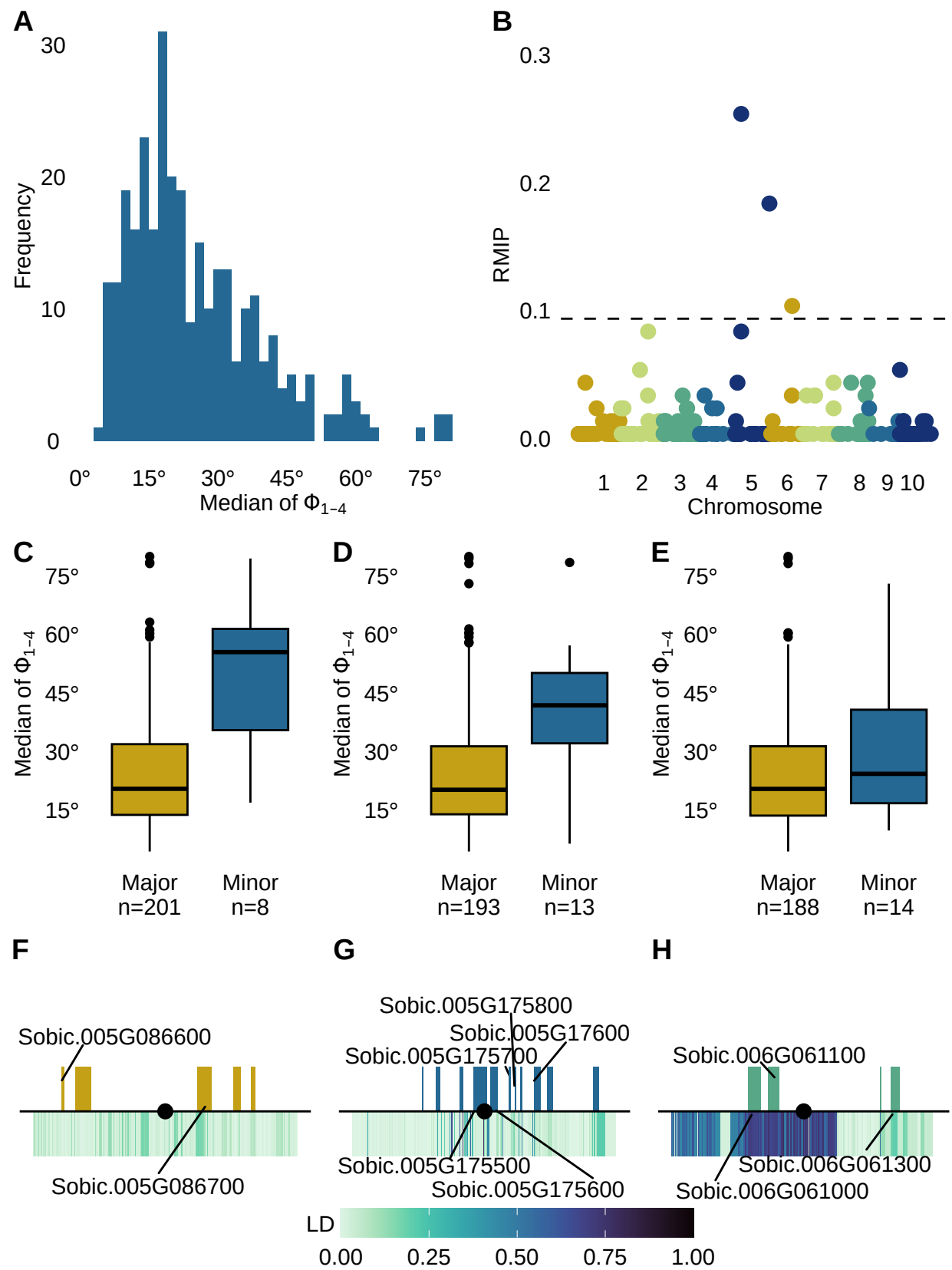

Supplement: Multimedia component 3 [file mmc3.zip › figs/figure6.pdf]

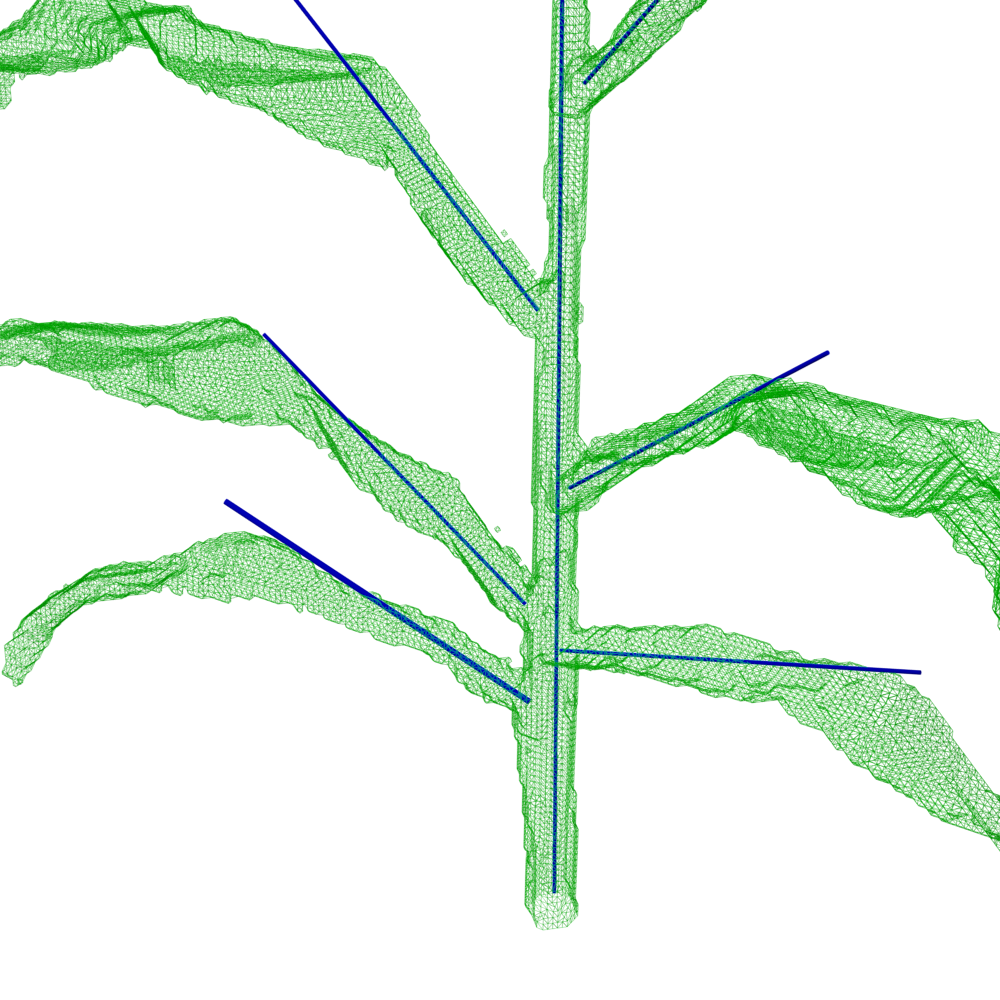

Supplement: Multimedia component 3 [file mmc3.zip › figs/fig_angle_rendering/fig_angle_rendering_165.png]

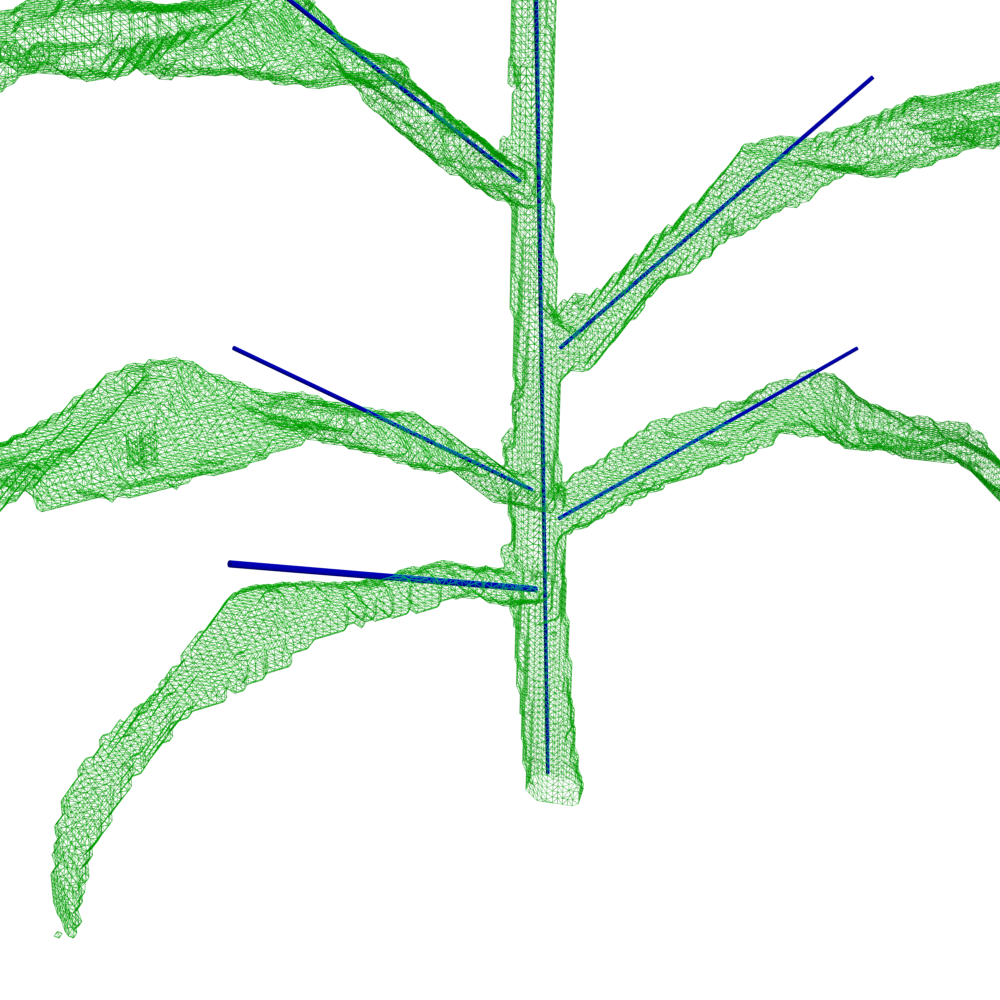

Supplement: Multimedia component 3 [file mmc3.zip › figs/fig_angle_rendering/fig_angle_rendering_0.png]

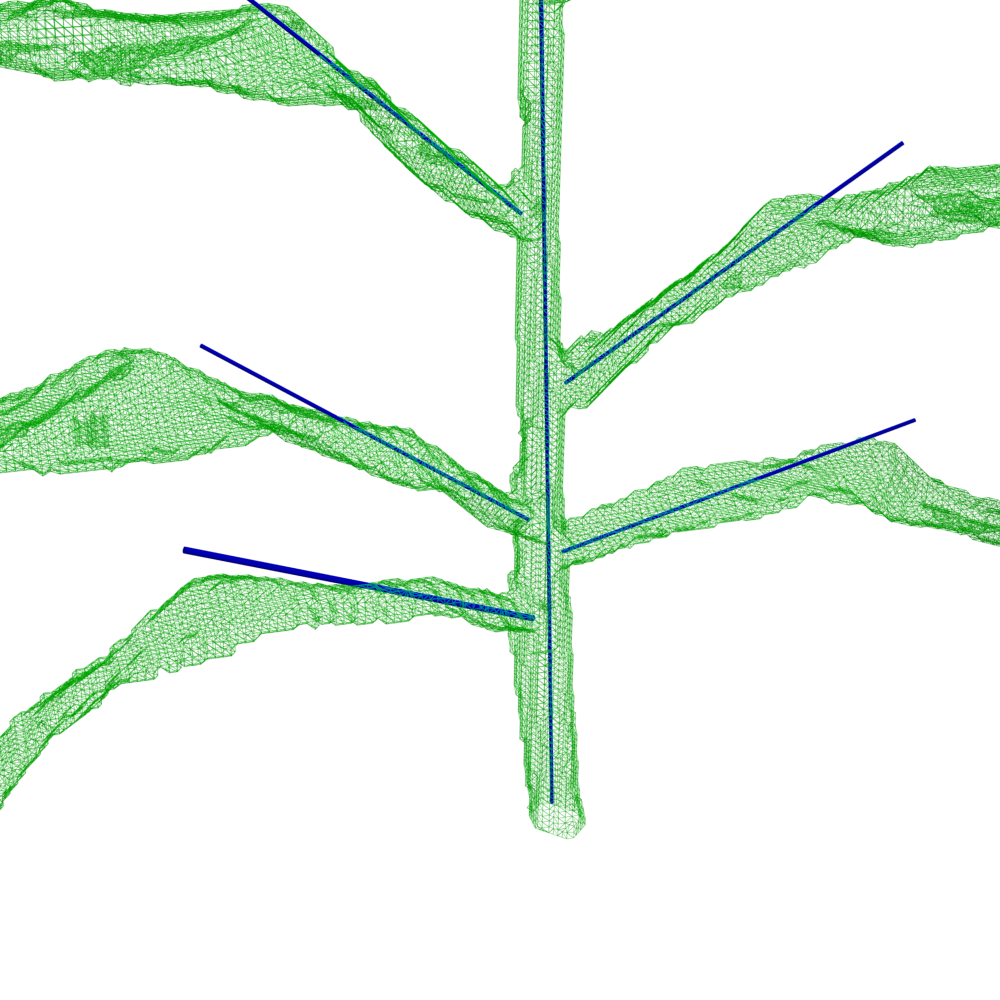

Supplement: Multimedia component 3 [file mmc3.zip › figs/fig_angle_rendering/fig_angle_rendering_80.png]

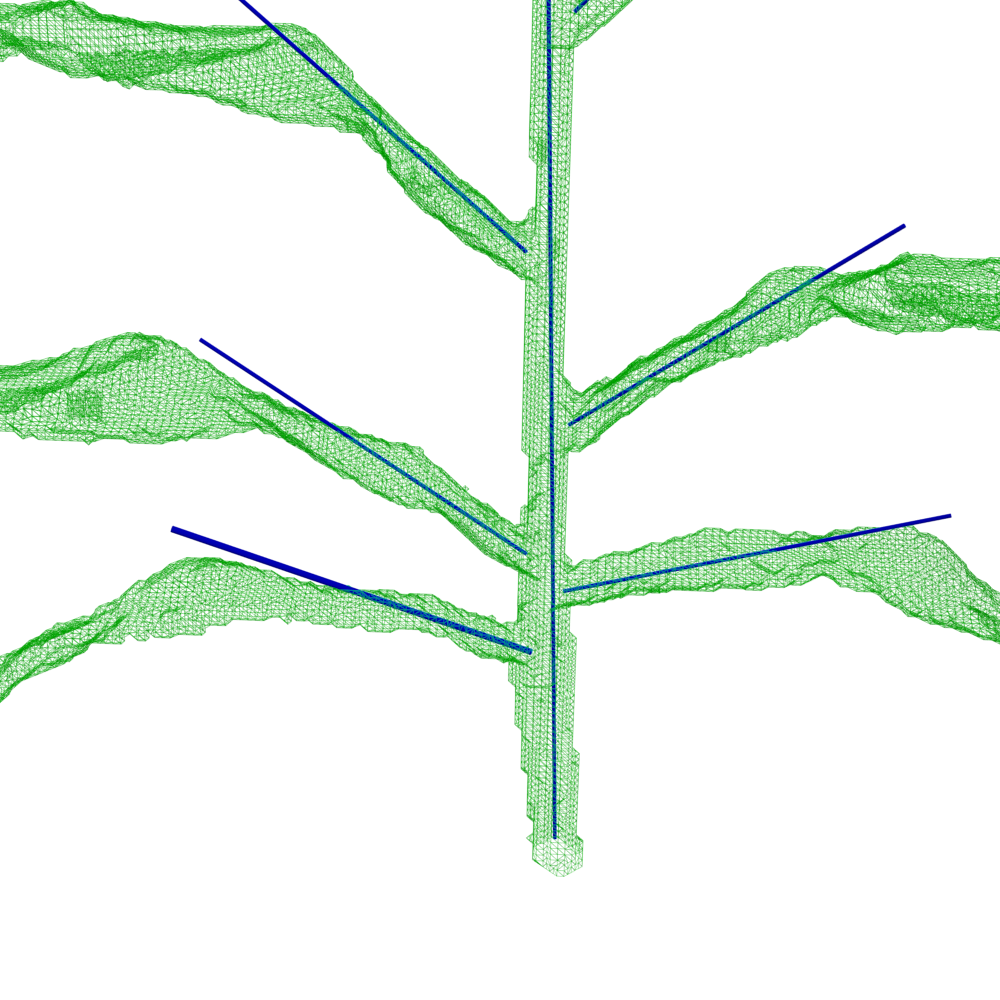

Supplement: Multimedia component 3 [file mmc3.zip › figs/fig_angle_rendering/fig_angle_rendering_120.png]
