# Supplementary material for: 3D reconstruction enables high-throughput phenotyping and quantitative genetic analysis of phyllotaxy
Source: Plant Phenomics. 2025 Mar 8;7(1):100023. doi: 10.1016/j.plaphe.2025.100023 (PMC12710043; doi:10.1016/j.plaphe.2025.100023)
Supplement: Multimedia component 3 [file mmc3.zip › figs/Workflow_Phyllotaxy.pdf]

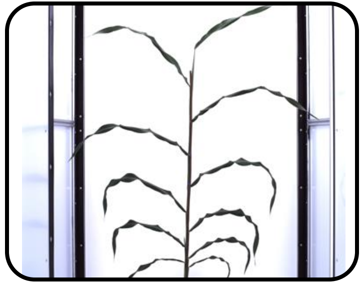

2D RGB Imaging

- 5 side views + 1 top view
- 366 plants
- 236 genotypes from SAP
- 3 timepoints

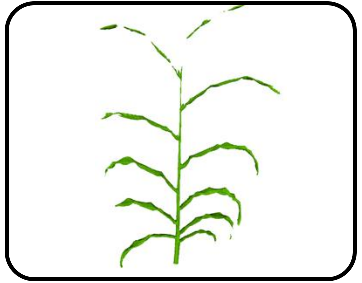

3D Reconstruction

- Image segmentation
- Voxel carving

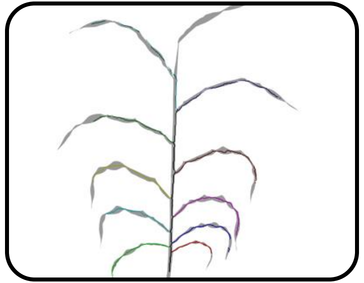

Skeletonization

- Thin voxels classified as leaves or stem
- Extract leaf position

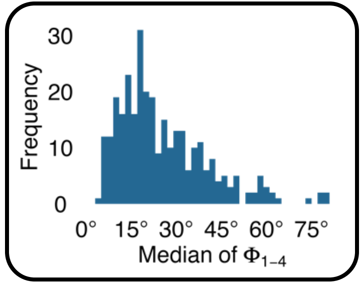

Data Processing

- Calculate difference of the angle between leaves from 180°
- Remove extreme values
- Summarize across timepoints

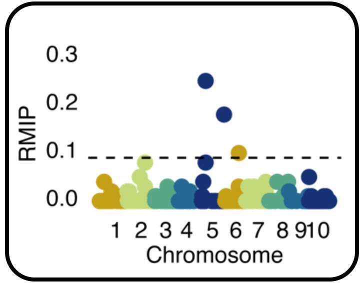

Quantitative Genetic Analysis

- Estimate heritability
- Resampling FarmCPU GWAS
